# Medication delivery and dispensing interval preferences of people who use antihypertensive medications in Australia: a survey study

**DOI:** 10.5694/mja2.70034

**Published:** 2025-08-28

**Authors:** Carissa Bonner, Michael A Fajardo, Rachael M Keast, Emily Atkins, Niamh Chapman, Kristie R Weir, Anthony Rodgers, Aletta E Schutte

**Affiliations:** ^1^ Leeder Centre for Health Policy, Economics and Data University of Sydney Sydney NSW; ^2^ The University of Sydney Sydney NSW; ^3^ The George Institute for Global Health Sydney NSW; ^4^ The University of New South Wales Sydney NSW

**Keywords:** Hypertension, Prescribing

Pharmacy‐related costs and fees comprise 51% of the total cost of hypertension medications in Australia, largely because of 30‐day dispensing periods, increasing out‐of‐pocket costs for patients.^1^ In 2022, the Pharmaceutical Benefits Advisory Committee (PBAC) recommended 60‐ or 90‐day dispensing periods for hypertension medications, a proposal supported by the Australian Hypertension Taskforce.^2^ The World Health Organization recommends 90‐day dispensing periods for improving medication adherence and long term blood pressure control.^3^ The recent move from 30‐ to 60‐day dispensing in Australia to reduce costs^4^, ^5^ has been controversial.^6^

We therefore explored the perspectives of hypertension medication purchasers in Australia to provide information that could inform dispensing policy. We investigated medication dispensing method preferences in an online survey codesigned with community panel members by the Sydney Health Literacy Lab.^7^ The design phase included iterative discussion, development, testing, and refinement by a group of seven men and women aged 30–85 years with differing cardiovascular risk and medications experience. Forced choice questions that compared different dispensing options were developed (Box 1). Estimated costs for people with or without concession card holders were based on Pharmaceutical Benefits Scheme (PBS) copayment thresholds and prices in May 2024 (Box 2).^8^

Box 1 Example of a forced choice binary question (choice 3 in Box 2) for people without and with concession cards*

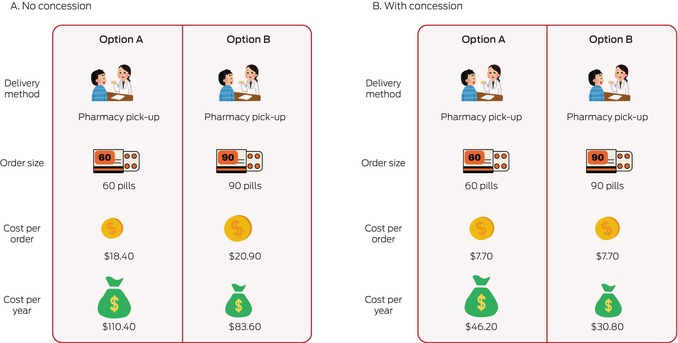

* The complete survey is included in the Supporting Information, part 1.

The participants in our survey were members of the Dynata market research panel (https://www.dynata.com), who receive points for completing a survey they could exchange for gift vouchers. Participation was anonymous, but we collected demographic characteristics, medication experience, and dispensing preference information. We used soft quota sampling to ensure diversity with respect to age (over or under 65 years of age), education (with or without university education), and gender (men or women) to recruit a national sample of 2000 adults (18 years or older) currently using blood pressure medications, sufficiently large to facilitate exploratory demographic subgroup analyses (Supporting Information, part 2). The Qualtrics survey (https://www.qualtrics.com) was available for online completion during 27 November 2024 – 21 January 2025. We summarise participant characteristics as descriptive statistics. We assessed the preferred mode of medication collection (pharmacy pickup or postal delivery) when the cost implications of choices were not provided in logistic multivariate regression models adjusted for reaching the Pharmaceutical Benefits Scheme (PBS) safety threshold, location (major city or other), preferred medication delivery duration, education level, gender, age, and income; we report adjusted odds ratios (aORs) with 95% confidence intervals (CIs). Analyses were undertaken in SPSS 29 (IBM). Free text responses were thematically coded. The University of Sydney Human Research Ethics Committee approved the study (2024/HE000957).

A total of 2054 people participated in the study; 912 (44%) were aged 65 years or older, 1014 (50%) were women, 1106 (54%) had university education, and 1576 (77%) lived in major cities. Reported weekly income was less than $1250 for 1121 people (55%) (Supporting Information, table 1), 1335 (65%) were concession card holders, and 1044 (51%) usually reached the PBS safety net each year. Systolic blood pressure in the range 130–144 mmHg was reported by 912 people (44%), 145 mmHg or higher by 404 (20%).

When costs implications were not included with the question, 1797 participants (89%) preferred pharmacy pickup and 234 (11%) postal delivery. Preference for pharmacy pickup was associated with not having a university education (aOR, 3.78; 95% CI, 2.57–5.57), not usually reaching the PBS safety net threshold (aOR, 1.95; 95% CI, 1.28–2.96), not having a concession card (aOR, 1.92; 95% CI, 1.23–3.01), not living in a major city (aOR, 1.65; 95% CI, 1.02–2.67), and preference for 30‐ or 60‐day dispensing period (aOR, 2.25; 95% CI, 1.64–3.09); it was more likely for men (aOR, 1.56; 95% CI, 1.14–2.15) and increased with age (per year: aOR, 1.03; 95% CI, 1.02–1.04), and was not influenced by income (aOR, 0.99; 95% CI, 0.93–1.05) (Supporting Information, table 2). Reasons provided in open responses for preferring pharmacy pickup included the opportunity for social interaction, exercise, and medical advice, and not trusting postal delivery. Reasons for preferring postal delivery included the time saving and convenience.

When the cost implications of the options were provided with the forced choice questions, most participants preferred pharmacy pickup to postal delivery (choices 1 and 2; 75–83% preferred pharmacy pickup). This pattern applied to people with or without concession cards, and for both 60‐ and 90‐day dispensing periods (Box 2; Supporting Information, figure 1).

When costs implications were not included with the question, 659 participants (32%) preferred 30‐day dispensing, 642 (31%) 60‐day dispensing, 413 (20%) 90‐day dispensing, and 56 (3%) 120‐day dispensing. Reasons for preferring shorter dispensing periods (30 or 60 days) included concerns about medicine quality, expiry, storage, and dose changes, and preferring the status quo. Reasons for preferring longer dispensing periods (60 or 120 days) included convenience, ease of remembering, and avoiding running out.

When the cost implications of the options were provided with the forced choice questions, most participants preferred 90‐day dispensing to 60‐day dispensing (choices 3 and 6; 69–92% preferred 90‐day dispensing) and 120‐day to 30‐day dispensing (choices 4 and 5; 83–92% preferred 120‐day dispensing). This pattern applied to people with or without concession cards, and for both delivery modes (pharmacy pickup and postal delivery) (Box 2; Supporting Information, figure 1).

Box 2 Forced choice binary questions about delivery mode and dispensing interval options, and responses to these questions when the cost implications of the choices were provided, by concession card status*

**Table:** 

Choice, with options	Delivery method	Dispensing interval (days)	Out‐of‐pocket cost, per dispensing	Out‐of‐pocket cost, per year	Respondents preferring this choice
Choice 1: pharmacy *v* postal (60‐day dispensing)					
Concession: option A	pharmacy	60	$7.70	$46.20	1100 (83%)
Concession: option B	postal	60	$7.70	$46.20	233 (17%)
No concession: option A	pharmacy	60	$18.40	$110.40	547 (76%)
No concession: option B	postal	60	$18.40	$110.40	170 (24%)
Choice 2: pharmacy *v* postal (90‐day dispensing)					
Concession: option A	pharmacy	90	$7.70	$30.80	1060 (80%)
Concession: option B	postal	90	$7.70	$30.80	274 (20%)
No concession: option A	pharmacy	90	$20.90	$83.60	536 (75%)
No concession: option B	postal	90	$20.90	$83.60	182 (25%)
Choice 3: 60‐ *v* 90‐day dispensing (pharmacy)					
Concession: option A	pharmacy	60	$7.70	$46.20	416 (31%)
Concession: option B	pharmacy	90	$7.70	$30.80	917 (69%)
No concession: option A	pharmacy	60	$18.40	$110.40	135 (19%)
No concession: option B	pharmacy	90	$20.90	$83.60	583 (81%)
Choice 4: 30‐ *v* 120‐day dispensing (postal)					
Concession: option A	postal	30	$7.70	$92.40	220 (17%)
Concession: option B	postal	120	$7.70	$23.10	1115 (83%)
No concession: option A	postal	30	$15.90	$190.80	60 (8%)
No concession: option B	postal	120	$23.40	$70.20	657 (92%)
Choice 5: 30‐ *v* 120‐day dispensing (pharmacy)					
Concession: option A	pharmacy	30	$7.70	$92.40	213 (16%)
Concession: option B	pharmacy	120	$7.70	$23.10	1119 (84%)
No concession: option A	pharmacy	30	$15.90	$190.80	57 (8%)
No concession: option B	pharmacy	120	$23.40	$70.20	659 (92%)
Choice 6: 60‐ *v* 90‐day dispensing (postal)					
Concession: option A	postal	60	$7.70	$46.20	214 (16%)
Concession: option B	postal	90	$7.70	$30.80	1117 (84%)
No concession: option A	postal	60	$18.40	$110.40	58 (8%)
No concession: option B	postal	90	$20.90	$83.60	658 (92%)

* These results are depicted graphically in the Supporting Information, figure 1. Further details by concession card status and demographic characteristics: Supporting Information, tables 3 and 4.

We found that a large majority of people who use hypertension medications prefer in‐person pickup at pharmacies to postal delivery, and longer dispensing intervals, when these choices are associated with lower out‐of‐pocket costs. Preferences differed by both demographic and behavioural factors; some participants, for example, saw broader social and health management benefits in pharmacy pickup. Some participants worried about the reliability of postal delivery methods, while others viewed postal delivery with longer dispensing periods as convenient and timesaving.

Limitations of our study include the use of an online market panel sample that may not be representative of all Australian adults who use hypertension medications. The gender and education characteristics of our participant group were similar to those of the adult Australian population,^9^ and the age distribution was similar to that for Australian adults with high blood pressure.^10^

Our survey findings will be used to guide a clinical trial that will investigate whether longer dispensing periods for anti‐hypertensive medications affect treatment adherence. They could also inform future decision making, given recent changes to permitted dispensing limits.

## Competing interests

Aletta Schutte has received speaker honoraria from Servier, Abbott, Sanofi, AstraZeneca, Medtronic, Omron, and Aktiia.

## Data sharing

Anonymous survey data will be available on request, subject to ethics approval.

## Author contributions

Conceptualisation: Carissa Bonner, Emily Atkins, Anthony Rodgers, Aletta Schutte. Data curation: Carissa Bonner, Michael Fajardo, Rachael Keast. Formal analysis: Michael Fajardo. Funding acquisition: Carissa Bonner, Anthony Rodgers, Aletta Schutte. Methodology: all authors. Project administration: Carissa Bonner, Rachael Keast. Writing (draft): Carissa Bonner. Writing (review and editing): all authors.

Received 2 March 2025, accepted 19 June 2025

## Supporting information


Supplementary methods and results
