# Supplementary material for: Medication delivery and dispensing interval preferences of people who use antihypertensive medications in Australia: a survey study
Source: Med J Aust. 2025 Aug 28;223(8):423–5. doi: 10.5694/mja2.70034 (PMC12536080; doi:10.5694/mja2.70034)
Supplement: Supplementary file 1 — Supplementary methods and results [file MJA2-223-423-s001.pdf]

MJA
The Medical Journal of Australia

Supporting Information

Supplementary methods and results

This appendix was part of the submitted manuscript and has been peer reviewed.
It is posted as supplied by the authors.

Appendix to: Bonner C, Fajardo MA, Keast RM, et al. Medication delivery and dispensing interval preferences of people who use antihypertensive medications in Australia: a survey study. *Med J Aust* 2025; doi: 10.5694/mja2.70034.

Supplementary methods

1. Survey text (insert: 16 pages)

Consent

About this survey

This study is a survey that aims to find out:

- Your experience of managing your blood pressure
- How you usually get medicines from a pharmacy (dispensing) once they are prescribed by a doctor
- Your preferences about how to get your medicine

The results from this survey will help us plan a trial to test different ways to access blood pressure medicines. To learn more about the study, please read the Participant Information Sheet.

How to complete this survey

Progress through the survey form by using the 'next' and 'previous' buttons at the bottom of each page. Do not use the browser forward and back buttons.

Please answer the questions as accurately as you can. If you do not know the exact answer, giving a close answer is still useful. For questions where you do not know a close answer, select 'I don't know'.

It should take roughly 15 minutes to complete survey. Once you have finished the survey simply click the 'Submit' button.

Please provide your consent to take part in this research by clicking the button below. If you do not wish to take part in this survey, close the browser window.

I have read and understood the participant information sheet provided

I understand that taking part in this research is voluntary and I provide my consent to take part in the survey

☐ I consent to participate

Eligibility Screen

Please answer the following questions to determine if you are eligible to take

part in this study.

Do you live in Australia?

- ◯ Yes
- ◯ No

What is your age category?

- ◯ Under 18 years
- ◯ 18 to 44 years
- ◯ 45 to 64 years
- ◯ 65 years or more

In the last two weeks, have you taken any medicine for high blood pressure?

- ◯ Yes
- ◯ No
- ◯ Don't know

Quota Sampling

Which term describes your gender identity?

- ◯ Male
- ◯ Female
- ◯ Trans and/or gender diverse
- ◯ ____________ I use a different term (please specify)
- ◯ I'd prefer not to say

What is your highest level of education?

- ◯ Below Year 10 (including Certificate I/II)
- ◯ Year 10 or equivalent (e.g. Leaving Certificate)
- ◯ Year 11
- ◯ Year 12 or equivalent (e.g. HSC, VCE)
- ◯ Certificate III/IV
- ◯ Diploma/Associate degree
- ◯ University degree (e.g. Bachelor degree)
- ◯ Graduate diploma or graduate certificate
- ◯ Postgraduate (e.g. Masters)

What is your postcode?

What is your year of birth?

Sample Characteristics

Section 1 of 3: About you

This section will help us work out if different groups have different preferences.

Are you a concession card holder?

- ◯ Yes
- ◯ No
- ◯ Don't know

Do you usually reach the Pharmaceutical Benefits Scheme (PBS) Safety Net in a calendar year?

- ◯ Yes
- ◯ No
- ◯ Don't know

Q. What is the usual weekly income of your household?

How many different kinds of prescription medicines do you take in a typical week (please include all types of medicines, not just blood pressure medicines)?

- ◯ 0
- ◯ 1 - 3
- ◯ 4 - 6
- ◯ 7 - 10
- ◯ 11 - 20
- ◯ More than 20

How many different kinds of non-prescription medicines do you take in a typical week? These include over-the-counter medicines, or herbal/natural supplements

and multivitamins.

- ◯ 0
- ◯ 1 - 3
- ◯ 4 - 6
- ◯ 7 - 10
- ◯ 11 - 20
- ◯ More than 20

Which method do you use most frequently to get your medicines?

- ◯ I receive medicines via postal or courier delivery
- ◯ I pick up medicines in-person at a pharmacy

Please rate how much you agree with the following statement: *I spend a lot of money on my medicines.*

- ◯ Strongly agree
- ◯ Agree
- ◯ Neither agree nor disagree
- ◯ Disagree
- ◯ Strongly disagree

In the last 12 months, have you or a health professional measured your blood pressure?

- ◯ Yes, I have measured my blood pressure
- ◯ Yes, a health professional has measured my blood pressure
- ◯ All of the above
- ◯ None of the above
- ◯ Don’t know

Has a health professional (e.g. doctor, nurse) ever told you that you have high blood pressure (hypertension)?

- ◯ Yes
- ◯ No
- ◯ Don't know

When you/your health professional measure your blood pressure, what is your reading typically?

Systolic blood pressure (larger top number)

Diastolic blood pressure (smaller bottom number)

Preferences

Section 2 of 3: Your preferences

These questions will help us understand your preferences about getting medicine.

Imagine your doctor has told you to take a new blood pressure medicine long term. They offer you several choices for how to obtain the medicines.

Considering how you normally pick up your medicines, how many months' worth of medicine would you prefer to get at a time?

- ◯ 1 month
- ◯ 2 months
- ◯ 3 months
- ◯ 4 months
- ◯ 5 months
- ◯ 6 months

Reasons for this choice:

What is your preferred delivery format?

- ◯ Pick up at a pharmacy
- ◯ Delivery by post or courier

Reasons for this choice:

Now imagine your doctor says they are participating in a research study to see which option helps people manage their blood pressure the best. If you choose to participate, you would receive your medicine in one of the following ways. The option will be chosen for you at random.

- Pick-up at your usual pharmacy, 1 month supply
- Pick-up at your usual pharmacy, 2 month supply
- Pick-up at your usual pharmacy, 3 month supply
- Postal delivery, 1 month supply
- Postal delivery, 2 month supply
- Postal delivery, 3 month supply
- Postal delivery, 4 month supply

◯ Yes
◯ No

Please explain the reason for your response:

Non-concession

Now imagine your doctor offers you different options for getting your blood pressure medicine.

Which ONE out of these two options would you prefer?

	OPTION A	OPTION B
Delivery method	Pharmacy pick-up	Postal delivery
Order size	60 pills	60 pills
Cost per order	$18.40	$18.40
Cost per year	$110.40	$110.40

◯ Option A
◯ Option B

Which ONE out of these two options would you prefer?

	OPTION A	OPTION B
Delivery method	Pharmacy pick-up	Postal delivery
Order size	90 pills	90 pills
Cost per order	$20.90	$20.90
Cost per year	$83.60	$83.60

◯ Option A
◯ Option B

Which ONE out of these two options would you prefer?

	OPTION A	OPTION B
Delivery method	Pharmacy pick-up	Pharmacy pick-up
Order size	60 pills	90 pills
Cost per order	$18.40	$20.90
Cost per year	$110.40	$83.60

- ◯ Option A
- ◯ Option B

Which ONE out of these two options would you prefer?

	OPTION A	OPTION B
Delivery method	Postal delivery	Postal delivery
Order size	30 pills	120 pills
Cost per order	$15.90	$23.40
Cost per year	$190.80	$70.20

◯ Option A
◯ Option B

Which ONE out of these two options would you prefer?

	OPTION A	OPTION B
Delivery method	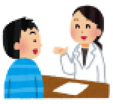 Pharmacy pick-up	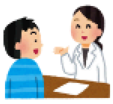 Pharmacy pick-up
Order size	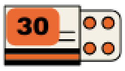 30 pills	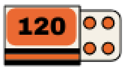 120 pills
Cost per order	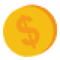 $15.90	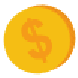 $23.40
Cost per year	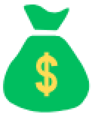 $190.80	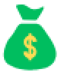 $70.20

◯ Option A
◯ Option B

Which ONE out of these two options would you prefer?

	OPTION A	OPTION B
Delivery method	Postal delivery	Postal delivery
Order size	60 pills	90 pills
Cost per order	$18.40	$20.90
Cost per year	$110.40	$83.60

◯ Option A
◯ Option B

Please indicate how affordable each of these costs would be for you to pay at once for medicine:

	1 = Not affordable	2	3	4	5 = Completely Affordable
$15.90 for 1 month supply	◯	◯	◯	◯	◯
$18.40 for 2 month supply	◯	◯	◯	◯	◯
$20.90 for 3 month supply	◯	◯	◯	◯	◯
$23.40 for 4 month supply	◯	◯	◯	◯	◯

Please indicate how affordable each of these costs would be for you to pay in a year for medicine:

	1 = Not affordable	2	3	4	5 = Completely affordable
$70.20 per year	◯	◯	◯	◯	◯
$83.60 per year	◯	◯	◯	◯	◯
$110.40 per year	◯	◯	◯	◯	◯
$190.80 per year	◯	◯	◯	◯	◯

Concession

Now imagine your doctor offers you different options for getting your blood pressure medicine.

Which ONE out of these two options would you prefer?

	OPTION A	OPTION B
Delivery method	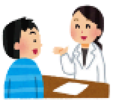 Pharmacy pick-up	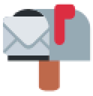 Postal delivery
Order size	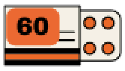 60 pills	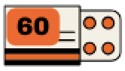 60 pills
Cost per order	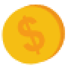 $7.70	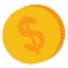 $7.70
Cost per year	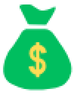 $46.20	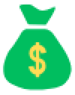 $46.20

◯ Option A
◯ Option B

Which ONE out of these two options would you prefer?

	OPTION A	OPTION B
Delivery method	Pharmacy pick-up	Postal delivery
Order size	90 pills	90 pills
Cost per order	$7.70	$7.70
Cost per year	$30.80	$30.80

◯ Option A
◯ Option B

Which ONE out of these two options would you prefer?

	OPTION A	OPTION B
Delivery method	Pharmacy pick-up	Pharmacy pick-up
Order size	60 pills	90 pills
Cost per order	$7.70	$7.70
Cost per year	$46.20	$30.80

◯ Option A

◯ Option B

Which ONE out of these two options would you prefer?

	OPTION A	OPTION B
Delivery method	Postal delivery	Postal delivery
Order size	30 pills	120 pills
Cost per order	$7.70	$7.70
Cost per year	$92.40	$23.10

◯ Option A
◯ Option B

Which ONE out of these two options would you prefer?

	OPTION A	OPTION B
Delivery method	Pharmacy pick-up	Pharmacy pick-up
Order size	30 pills	120 pills
Cost per order	$7.70	$7.70
Cost per year	$92.40	$23.10

◯ Option A
◯ Option B

Which ONE out of these two options would you prefer?

	OPTION A	OPTION B
Delivery method	Postal delivery	Postal delivery
Order size	60 pills	90 pills
Cost per order	$7.70	$7.70
Cost per year	$46.20	$30.80

◯ Option A

○ Option B

Please indicate how affordable each of these costs would be for you to pay at once for medicine:

	1= Not affordable	2	3	4	5 = Completely affordable
$7.70 for 1 month supply	○	○	○	○	○
$7.70 for 2 month supply	○	○	○	○	○
$7.70 for 3 month supply	○	○	○	○	○
$7.70 for 4 month supply	○	○	○	○	○

Please indicate how affordable each of these costs would be for you to pay in a year for medicine:

	1 = Not affordable	2	3	4	5 = Completely affordable
$23.10 per year	○	○	○	○	○
$30.80 per year	○	○	○	○	○
$46.20 per year	○	○	○	○	○
$92.40 per year	○	○	○	○	○

Experiences

Section 3 of 3: Your experience

This last section is about your experience with blood pressure medicines.

How many different **blood pressure** medicines do you take per day? We are interested in how many different *types* of blood pressure medicines you take each day, not the number of tablets you take.

○ 1
○ 2
○ 3
○ 4
○ 5
○ More than 5
○ Not sure

Each time you pick up your blood pressure medicines, how many months does this supply usually last?

[ ] months

How much do you usually pay each time you pick up your blood pressure medicines (after Medicare rebate or other subsidies)?

$ [ ]

How many repeats are usually on the prescription for your blood pressure medicine?

[ ]

To get a new blood pressure medicine prescription, who do you usually need to see?Please select all that apply

- [ ] GP – in person
- [ ] GP – online or phone consultation
- [ ] Nurse
- [ ] Pharmacist
- [ ] Cardiologist
- [ ] [ ] Other

How much do you usually pay out of pocket for the appointment to get a new blood pressure medicine prescription?

$ [ ]

Please list all the names of all the medicines you take regularly (please include all types of medicines for this question, not just blood pressure medicines):

[ ]

Is there anything else you would like to tell us about how you would like to get your blood pressure medicine?

[ ]

2. Survey participant recruitment

The target sample size was 2000 participants, based on the project budget and previous surveys using the same recruitment method which found that this number enabled sufficient diversity to explore and identify group differences related to health preferences; that is, no statistical power calculation was undertaken. Recruitment was conducted by Dynata from their online market research panel. A Qualtrics survey was set up with self-reported eligibility questions, were being an adult (18 years or older) in Australia taking blood pressure medication. Demographic questions were used for the quota sampling function in Qualtrics, based on equal recruitment of male/female, younger/middle/older age groups, and no university education/university education categories. When a specific gender/age/education category was filled (i.e. enough participants had completed the survey in the category given the aim of 2000 participants), no further recruitment in that group was conducted. The quotas were relaxed after reaching 20% in the 18-44 year old category (given lower prevalence of high blood pressure in this age group, this was the hardest group to recruit). Recruitment closed after 2000 eligible participants had provided consent, and the final sample was 2054 once all surveys were completed. As this was a descriptive, exploratory survey to inform the design of a trial, we did not pre-specify a power calculation. The trial will be registered with a pre-specified power calculation.

Supplementary results

Table 1. Study sample demographic characteristics, compared with the 2021 national population

Characteristic	Survey participants	Soft quota aim for subgroup analyses	Australian population
**Age group[1‡]**			
18 to 44 years*	449 (21.9%)	33.3%	42.4%
45 to 64 years	693 (33.7%)	33.3%	26.1%
65 years or more	912 (44.4%)	33.3%	18.3%
**Gender identity[1]**			
Male	1033 (50.4%)	50.0%	49.3%
Female	1014 (49.5%)	50.0%	50.7%
Trans and/or gender diverse	0	-	-
I use a different term (please specify)	1 (<0.1%)	-	-
I'd prefer not to say	2 (0.1%)	-	-
**Education (dichotomised)[2]**			
Below university degree	1106 (54.0%)	50.0%	37.0%
University degree or higher	944 (46.0%)	50.0%	63.0%
**Major city[3]**			
Living in a major city	1576 (77.4%)	-	66.9%
Does not live in a major city	459 (22.6%)	-	33.1%
**Weekly income[4†]**			
No income	40 (1.9%)	-	8.7%
Less than $1000	793 (37.9%)	-	49.1%
$1000 or more	1262 (60.2%)	-	41.5%

*The younger age group quota was relaxed after reaching 20% due to lower prevalence of blood pressure medication in this age group. A survey of people with hypertension finds a reversal in the age pattern from the general population, where 79% of people with hypertension were over 65 years pf age.

‡18–44 age group includes 14–17 years based on pooled ABS categories and the study sample is primarily blood pressure users, so it is not a direct comparison.

†ABS data is for 15 years old and over; percentages do not add up to 100 because the 'negative income' category was not included.

Note: Non-binary sex data was considered unreliable in the 2021 census.[6]

Table 2. Preference for pharmacy pickup (*v* postal delivery), when cost implications are not provided: logistic regression analysis, cost implications not provided

Characteristic	Raw responses	aOR (95% CI)*
Reaches PBS threshold (v does not reach threshold)†	Reaches: 983 (56.0%)<br>Does not reach 771 (44.0%)	1.95 (1.28-2.96)
No concession card (v concession card holder)	Concession Card: 1171 (66.8%)<br>No concession card: 583 (33.2%)	1.92 (1.23-3.01)
Does not live in major city (v major city)	Does not: 395 (22.5%)<br>Lives in major city: 1359 (77.5%)	1.65 (1.02-2.67)
Preferred 30-60 dispensing (v 90-180 day)	30-60 days: 1130 (64.4%)<br>90-180 days: 624 (35.6%)	2.25 (1.64-3.09)
No university education (university education)	University: 822 (46.9%)<br>No university: 932 (53.1%)	3.78 (2.57-5.57)
Gender (women v men)	Men: 884 (50.4%)<br>Women: 870 (49.6%)	1.56 (1.14-2.15)
Age (per year), mean (SD)	58.8 (16.4)	1.03 (1.02-1.04)
Weekly Income (per income category), mean (SD)‡	9.3 (3.2)	0.99 (0.93-1.05)
**Constant**		1.15

*Adjusted for reaching the Pharmaceutical Benefits Scheme (PBS) safety threshold, location (major city or other), preferred medication delivery format and duration, education level, gender, age, and income. Missing data: concession card, eight; major city, 19; frequency of delivery by preferred months, one; education, four; gender, seven (neither male nor female, three); age, 37; weekly income, 13; preferred delivery, 23. Final analysis sample for this logistic regression: 1773 participants.

† Reaches PBS threshold: 200 "don't know" responses excluded.

‡ Weekly income treated as continuous, given the ordinal nature of its collection and spread of results. Discrete continuous categories were in sequential order (0-14): No income, $1–149, $150–299, $300–399, $400–499, $500–649, $650–799, $800–999, $1000–1249, $1250–1499, $1500–1749, $1750–1999, $2000–2999 and $3000+.

**Model Summary**		
-2 Log likelihood	Cox & Snell R Square	Nagelkerke R Square
1065.678[a]	.124	.237

**Hosmer and Lemeshow Test**		
Chi-square	df	Sig.
7.091	8	.527

Figure 1. Forced choice binary questions about delivery mode and dispensing interval options, and responses to these questions when the cost implications of the choices were provided, by concession card status

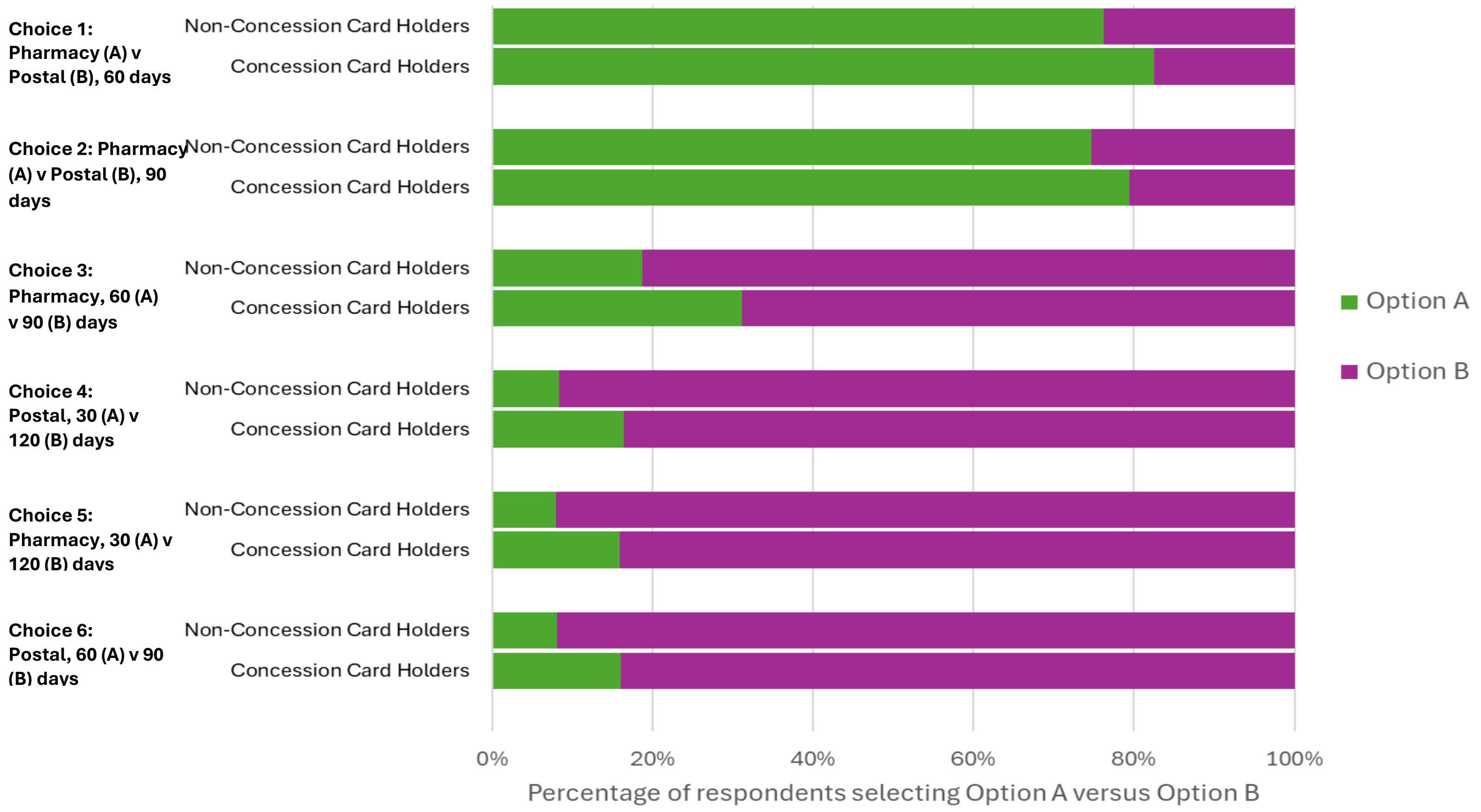

Table 3. Numbers and row proportions of preferences by sex, age group, location, PBS safety net, education, with cost implications of decisions provided: not concession card holders

	Choice 1				Choice 2				Choice 3				Choice 4				Choice 5				Choice 6			
	A		B		A		B		A		B		A		B		A		B		A		B	
**Characteristic**	N	%	N	%	N	%	N	%	N	%	N	%	N	%	N	%	N	%	N	%	N	%	N	%
**Gender**																								
Men	295	78	81	22	293	78	83	22	65	17	311	83	31	8	345	92	33	9	342	91	33	9	342	91
Women	247	74	89	26	239	71	98	29	68	20	269	80	28	8	308	92	23	7	313	93	23	7	313	93
**Age group**																								
18-44	140	66	71	34	132	63	79	37	42	20	169	80	27	13	184	87	25	12	186	88	29	14	182	86
45-65	253	77	74	23	250	76	78	24	62	19	266	81	27	8	301	92	26	8	300	92	25	8	302	92
65+	154	86	25	14	154	86	25	14	31	17	148	83	6	3	172	97	6	3	173	97	4	2	174	98
**Major city**																								
Yes	444	75	147	25	431	73	160	27	110	19	481	81	50	8	541	92	49	8	540	92	51	9	539	91
No	97	82	22	18	99	83	20	17	22	18	97	82	8	7	110	93	7	6	112	94	6	5	112	95
**PBS safety net threshold reached**																								
Yes	95	77	28	23	89	72	34	28	29	24	94	76	25	20	98	80	22	18	101	82	21	17	102	83
No	382	77	117	23	378	76	122	24	92	18	408	82	32	6	467	94	33	7	465	93	34	7	464	93
**Education**																								
University degree or higher	267	73	100	27	260	71	108	29	66	18	302	82	33	9	334	91	36	10	332	90	38	10	329	90
Below University degree	279	80	70	20	275	79	74	21	68	19	281	81	26	7	323	93	20	6	327	94	19	5	329	95

Table 4. Numbers and row proportions of preferences by sex, age group, location, PBS safety net, education, with cost implications of decisions provided: concession card holders

	Choice 1				Choice 2				Choice 3				Choice 4				Choice 5				Choice 6			
	A		B		A		B		A		B		A		B		A		B		A		B	
**Characteristic**	**N**	**%**	**N**	**%**	**N**	**%**	**N**	**%**	**N**	**%**	**N**	**%**	**N**	**%**	**N**	**%**	**N**	**%**	**N**	**%**	**N**	**%**	**N**	**%**
**Gender**																								
Men	559	85	97	15	539	82	117	18	221	34	434	66	106	16	550	84	118	18	0	82	113	17	540	83
Women	539	80	136	20	519	77	157	23	195	29	482	71	114	17	563	83	94	14	0	86	101	15	575	85
**Age group**																								
18-44	175	74	63	26	158	66	80	34	92	39	145	61	75	32	163	69	70	30	0	71	75	32	163	69
45-65	270	74	93	26	250	69	114	31	112	31	253	69	74	20	291	80	78	21	0	79	75	21	289	79
65+	655	90	77	10	652	89	80	11	212	29	519	71	71	10	661	90	65	9	0	91	64	9	665	91
**Major city**																								
Yes	798	81	185	19	765	78	219	22	320	33	663	67	190	19	795	81	184	19	0	81	189	19	793	81
No	295	87	44	13	288	85	51	15	91	27	248	73	24	7	315	93	24	7	0	93	22	7	317	94
**PBS safety net threshold reached**																								
Yes	741	81	177	19	711	77	208	23	337	37	581	63	192	21	728	79	187	20	0	80	195	21	724	79
No	270	87	40	13	260	84	50	16	53	17	257	83	22	7	288	93	22	7	0	93	14	5	295	96
**Education**																								
University degree or higher	433	76	140	24	409	71	165	29	213	37	361	63	151	26	424	74	148	26	0	74	154	27	418	73
Below University degree	664	88	93	12	648	86	109	14	203	27	553	73	68	9	689	91	65	9	0	91	60	8	696	92

References

1 Australian Bureau of Statistics. Population: Census 2021 https://www.abs.gov.au/statistics/people/population/population-census/2021 (viewed May 2025)

2 Australian Bureau of Statistics. Education and Work, Australia 2024 https://www.abs.gov.au/statistics/people/education/education-and-work-australia/may-2024 (viewed May 2025)

3 Australian Bureau of Statistics. Location: Census 2021 https://www.abs.gov.au/statistics/people/people-and-communities/location-census/2021 (viewed May 2025)

4 Australian Bureau of Statistics. Income and work: Census 2021 https://www.abs.gov.au/statistics/labour/earnings-and-working-conditions/income-and-work-census/2021 (viewed May 2025)

5 Australian Bureau of Statistics. Hypertension and high measured blood pressure 2022 https://www.abs.gov.au/statistics/health/health-conditions-and-risks/hypertension-and-high-measured-blood-pressure/latest-release (viewed May 2025)

6 Australian Bureau of Statistics. Analysis of non-binary sex responses 2022 https://www.abs.gov.au/articles/analysis-non-binary-sex-responses (viewed May 2025)

Checklist for Reporting Of Survey Studies (CROSS)

Note: The page numbers in this checklist refer to the submitted manuscript, not to the published article or its Supporting Information file

Section/topic	Item	Item description	Reported on page #
**Title and abstract**			
Title and abstract	1a	State the word "survey" along with a commonly used term in title or abstract to introduce the study's design.	2
	1b	Provide an informative summary in the abstract, covering background, objectives, methods, findings/results, interpretation/discussion, and conclusions.	N/A for research letter
**Introduction**			
Background	2	Provide a background about the rationale of study, what has been previously done, and why this survey is needed.	3
Purpose/aim	3	Identify specific purposes, aims, goals, or objectives of the study.	3
**Methods**			
Study design	4	Specify the study design in the methods section with a commonly used term (e.g., cross-sectional or longitudinal).	3
	5a	Describe the questionnaire (e.g., number of sections, number of questions, number and names of instruments used).	3, Fig 1, Appendix
Data collection methods	5b	Describe all questionnaire instruments that were used in the survey to measure particular concepts. Report target population, reported validity and reliability information, scoring/classification procedure, and reference links (if any).	3, Fig 1, Appendix
	5c	Provide information on pretesting of the questionnaire, if performed (in the article or in an online supplement). Report the method of pretesting, number of times questionnaire was pre-tested, number and demographics of participants used for pretesting, and the level of similarity of demographics between pre-testing participants and sample population.	3
	5d	Questionnaire if possible, should be fully provided (in the article, or as appendices or as an online supplement).	Appendix
Sample characteristics	6a	Describe the study population (i.e., background, locations, eligibility criteria for participant inclusion in survey, exclusion criteria).	3, Table S1
	6b	Describe the sampling techniques used (e.g., single stage or multistage sampling, simple random sampling, stratified sampling, cluster sampling, convenience sampling). Specify the locations of sample participants whenever clustered sampling was applied.	3, Table S1 notes
	6c	Provide information on sample size, along with details of sample size calculation.	3
	6d	Describe how representative the sample is of the study population (or target population if possible), particularly for population-based surveys.	3, Table S1
Survey administration	7a	Provide information on modes of questionnaire administration, including the type and number of contacts, the location where the survey was conducted (e.g., outpatient room or by use of online tools, such as SurveyMonkey).	3
	7b	Provide information of survey's time frame, such as periods of recruitment, exposure, and follow-up days.	3
	7c	Provide information on the entry process:<br>–>For non-web-based surveys, provide approaches to minimize human error in data entry.<br>–>For web-based surveys, provide approaches to prevent "multiple participation" of participants.	3
Study preparation	8	Describe any preparation process before conducting the survey (e.g., interviewers' training process, advertising the survey).	3
Ethical considerations	9a	Provide information on ethical approval for the survey if obtained, including informed consent, institutional review board [IRB] approval, Helsinki declaration, and good clinical practice [GCP] declaration (as appropriate).	3
	9b	Provide information about survey anonymity and confidentiality and describe what mechanisms were used to protect unauthorized access.	3
Statistical analysis	10a	Describe statistical methods and analytical approach. Report the statistical software that was used for data analysis.	3-4
	10b	Report any modification of variables used in the analysis, along with reference (if available).	N/A

	10c	Report details about how missing data was handled. Include rate of missing items, missing data mechanism (i.e., missing completely at random [MCAR], missing at random [MAR] or missing not at random [MNAR]) and methods used to deal with missing data (e.g., multiple imputation).	Tables
	10d	State how non-response error was addressed.	N/A
	10e	For longitudinal surveys, state how loss to follow-up was addressed.	N/A
	10f	Indicate whether any methods such as weighting of items or propensity scores have been used to adjust for non-representativeness of the sample.	N/A
	10g	Describe any sensitivity analysis conducted.	N/A
**Results**			
Respondent characteristics	11a	Report numbers of individuals at each stage of the study. Consider using a flow diagram, if possible.	N/A
	11b	Provide reasons for non-participation at each stage, if possible.	N/A
	11c	Report response rate, present the definition of response rate or the formula used to calculate response rate.	N/A
	11d	Provide information to define how unique visitors are determined. Report number of unique visitors along with relevant proportions (e.g., view proportion, participation proportion, completion proportion).	N/A
Descriptive results	12	Provide characteristics of study participants, as well as information on potential confounders and assessed outcomes.	4-5, Table S1
Main findings	13a	Give unadjusted estimates and, if applicable, confounder-adjusted estimates along with 95% confidence intervals and p-values.	4-5
	13b	For multivariable analysis, provide information on the model building process, model fit statistics, and model assumptions (as appropriate).	3
	13c	Provide details about any sensitivity analysis performed. If there are considerable amount of missing data, report sensitivity analyses comparing the results of complete cases with that of the imputed dataset (if possible).	N/A
**Discussion**			
Limitations	14	Discuss the limitations of the study, considering sources of potential biases and imprecisions, such as non-representativeness of sample, study design, important uncontrolled confounders.	7
Interpretations	15	Give a cautious overall interpretation of results, based on potential biases and imprecisions and suggest areas for future research.	5
Generalizability	16	Discuss the external validity of the results.	5
**Other sections**			
Role of funding source	17	State whether any funding organization has had any roles in the survey's design, implementation, and analysis.	1
Conflict of interest	18	Declare any potential conflict of interest.	1
Acknowledgements	19	Provide names of organizations/persons that are acknowledged along with their contribution to the research.	1
